# International validation of a pre-transplant risk assessment tool for graft survival in pediatric kidney transplant recipients

**DOI:** 10.1093/ckj/sfaf031

**Published:** 2025-01-28

**Authors:** Loes Oomen, Liesbeth L de Wall, Burkhard Tönshoff, Kai Krupka, Jerome Harambat, Julien Hogan, Cécile Couchoud, Emilie Savoye, Huib de Jong, Elisabeth A M Cornelissen, Antonia H M Bouts, Mandy G Keijzer-Veen, Wout F J Feitz, Charlotte M H H T Bootsma-Robroeks

**Affiliations:** Department of Urology, Division of Paediatric Urology, Radboudumc Amalia Children's Hospital, Nijmegen, The Netherlands; Department of Urology, Division of Paediatric Urology, Radboudumc Amalia Children's Hospital, Nijmegen, The Netherlands; CERTAIN Registry, Department of Paediatrics I, University Children's Hospital Heidelberg, Heidelberg, Germany; CERTAIN Registry, Department of Paediatrics I, University Children's Hospital Heidelberg, Heidelberg, Germany; Department of Pediatrics, Pediatric Nephrology Unit, Bordeaux University Hospital, Bordeaux, France; Department of Pediatric Nephrology and Transplantation, Robert-Debré Hospital, APHP, Paris, France; CRISTAL Registry, Agence de La Biomédicine, Paris, France; CRISTAL Registry, Agence de La Biomédicine, Paris, France; Department of Paediatric Nephrology, Erasmus MC-Sophia Children's Hospital, Rotterdam, The Netherlands; Department of Paediatric Nephrology, Radboudumc Amalia Children's Hospital, Nijmegen, The Netherlands; Department of Paediatric Nephrology, Amsterdam University Medical Centre, Emma Children's Hospital, Amsterdam, The Netherlands; Department of Paediatric Nephrology, Wilhelmina Children's Hospital, University Medical Centre Utrecht, Utrecht, The Netherlands; Department of Urology, Division of Paediatric Urology, Radboudumc Amalia Children's Hospital, Nijmegen, The Netherlands; Department of Paediatric Nephrology, Radboudumc Amalia Children's Hospital, Nijmegen, The Netherlands; University of Groningen, University Medical Centre Groningen, Department of Paediatrics, Paediatric Nephrology, Beatrix Children's Hospital, Groningen, The Netherlands

**Keywords:** graft survival, pediatric kidney transplantation, prediction model, validation study

## Abstract

**Background:**

A pre-transplant prediction model using commonly available factors is valuable for optimizing donor selection, communication, and counseling for pediatric kidney transplant (PKT) recipients. This study aims to externally validate a Dutch PKT prediction model and assess its international applicability.

**Materials and methods:**

Data from the Dutch-, CERTAIN-, and CRISTAL registries, covering PKT from 2005 to 2021, were used. The Dutch prediction model was externally validated in a German and French cohort and then adapted to these specific countries. An international prediction model was also developed using all available data. Models were based on 80% derivation cohorts and internally validated using areas under the receiver operating characteristic curve (ROC-AUC) and calibration plots.

**Results:**

Of 3266 transplantations, 2475 (273 Dutch, 356 German, 1622 French, and 224 other) were used for analysis. Cohorts differed significantly in baseline characteristics and outcomes. Internal validation of the Dutch model showed ROC-AUC of 0.77 and 0.75 at 10 and 15 years. External validation in German and French cohorts yielded 10-year ROC-AUC of 0.63 and 0.60, respectively.

Internal validation of the international prediction model showed AUC of 0.61 and 0.60 at 10 and 15 years with poor calibration, indicating inferior performance. The adapted national models showed better internal validation performance, with 10-year ROC-AUC of 0.77, 0.76, and 0.73 in Dutch, French, and German cohorts, respectively.

**Conclusions:**

The Dutch PKT prediction tool requires country-specific adaptations for use in other countries, given the diversity of clinical practice across Europe. A country-specific model is preferable to an international model in the current landscape.

## Graphical Abstract

**Graphical Abstract:**
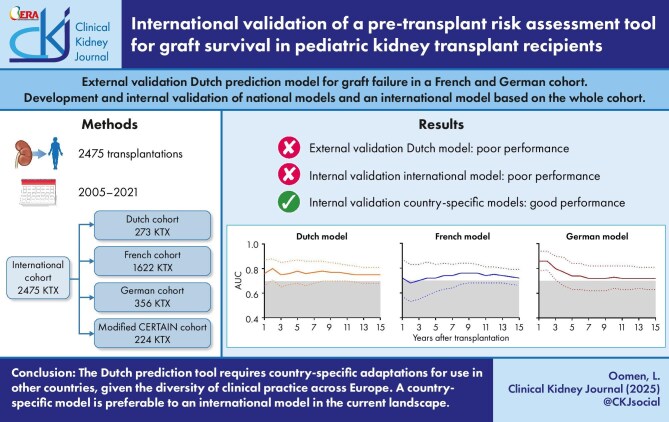

KEY LEARNING POINTS **What was known**:

- The outcome of kidney transplantation is influenced by an interplay of multiple factors.
- Predicting graft survival prior to transplantation can help in determining the optimal transplantation strategy.
- A Dutch pre-transplant prediction model for graft survival after pediatric kidney transplantation has demonstrated good predictive performance in the Netherlands.

**This study adds**:

- These predictive models only work in their own countries.
- External validation of the Dutch model showed poor predictive performance, requiring adaptations for international use.
- Similarly, a generalized international model based on 2475 European transplants performed poorly, likely due to country heterogeneity.
- However, country-specific models performed well in their own country.

**Potential impact**:

- Country-specific prediction models show promise in supporting clinical decision-making and personalized medicine by comparing different scenarios for individual patients.
- Differences among countries hinder international models, presenting an opportunity for future research to learn from each other and potentially develop unified guidelines.

## INTRODUCTION

Pediatric kidney transplantation (PKT) is the preferred treatment for children with kidney failure (stage 5 chronic kidney disease), and continuous advances have significantly improved both patient and graft survival outcomes over time [1, 2]. However, there is still room for improvement in long-term outcomes, and individualized prediction of long-term allograft failure would facilitate clinical decision-making and patient counseling.

Transplantation success involves a complex interplay of several factors [3–7]. Although several pre-transplant parameters are known to independently influence graft survival, their relative weights and combined impact remain relatively unexplored [4, 8, 9]. Besides, the relative hazard of these parameters is not constant over time, as described by Coens *et al.* [10]. To facilitate optimal donor selection for PKT recipients, a pre-transplant prediction model incorporating only commonly available pre-transplant factors could be of great value.

Over time, several models have been developed to predict graft survival. Yet, these models either rely on post-transplantation data or lack specificity for the pediatric population [11–13]. Therefore, we developed a pediatric pre-transplant risk assessment tool based on data from all PKTs in the Netherlands between 1990 and 2021. Through internal validation, the tool has demonstrated good prediction performance for graft survival in Dutch PKT patients [14].

The care of PKT recipients in the Netherlands may differ from that in other (European) countries. To ensure wider applicability of the model, it is important to assess and confirm its reproducibility and generalizability across Europe.

In addition, given the potential requirements for adapting this model to other populations, it is worth exploring whether the predictive accuracy of such a model benefits from international data or whether a country-specific approach is preferable.

Therefore, the primary objective of this study was to perform an external validation of the Dutch risk assessment tool.

The secondary objective was to develop and evaluate an international prediction model and to determine whether an international or a country-specific approach is more accurate.

## MATERIALS AND METHODS

### Original model and dataset

Our original risk assessment tool was based on data from the Dutch organ transplantation registry NOTR (Nederlandse Orgaan Transplantatie Registratie; Dutch Organ Transplantation Registry) as described previously [14].

In brief, this model was based on 554 PKTs from 1990 to 2021. To develop this model, a multivariable binary logistic regression analysis was performed, including the following parameters: recipient age, donor age, living donor (LD) status, pre-emptive transplantation, re-transplant, number of human leukocyte antigen (HLA) mismatches, number of HLA-DR mismatches, and the underlying primary disease responsible for kidney failure. These were classified as congenital anomalies of the kidney and urinary tract (CAKUT), ciliopathy, glomerulopathy, metabolic nephropathy, rare cause of hypertension, tubulopathy, or other. The model was adjusted to account for the decade (era) in which the transplantations took place (Appendix 2).

Variable selection was knowledge driven: incorporating findings from literature reviews and expert opinions to identify potential predictors of graft function. All selected predictors were readily available clinical parameters known prior to transplantation. Variables such as sex mismatches, duration of dialysis, HLA-DQ mismatches, and donor health were excluded due to missing data, while ABO incompatibility was excluded because of its low incidence.

A person-period file was constructed, with each month of follow-up treated as an individual observation point. Multivariate binary logistic regression was performed, and the β-regression coefficients from this analysis were used to develop a prediction model. These β-coefficients served as slope coefficients in the prediction formula that calculated the hazard of graft failure for each patient at a given time point after transplantation. These hazard estimates were then used to compute predicted survival, which facilitated the creation of individual survival curves based on the available parameters.

This predictive model enables clinicians to estimate monthly graft survival in various clinical scenarios using pre-transplantation data. By allowing comparisons between different donors for a specific patient, it supports personalized clinical decision-making.

### Data collection for external validation

An independent geographical validation approach was used to test the Dutch prediction model in a set of international patient data [15, 16]. The data for this external validation were derived from the Cooperative European Paediatric Renal Transplant Initiative (CERTAIN) registry [17] and the French CRISTAL [18] registry.

The web-based CERTAIN registry, launched in 2011, serves as a research platform for PKT and collects comprehensive longitudinal data on recipients under the age of 21 years at the time of transplantation from 95 actively participating centers in 26 countries [17, 19]. Enrollment in the CERTAIN registry requires the approval of the ethics committee of each participating center. Informed consent from parents or legal guardians is required before patients can be enrolled, and patients are required to give consent if appropriate for their age [17, 20]. Data are collected at several time points: baseline (pre-transplant), 1, 3-, 6-, 9-, and 12-months post-transplant, and every 6 months thereafter. Data in this database are collected retrospectively starting from 1991, with prospective data collection implemented since 2011.

CRISTAL is a mandatory national database, initiated in 1996, driven by the Agence de la biomédecine [18, 21]. Data on all organ transplantation candidates, recipients, and donors in France are collected prospectively by each transplantation center. Demographics, clinical, and laboratory data are collected at several key points, such as listing, transplantation surgery, and at subsequent annual intervals until the patient's death.

Follow-up data were used until either graft loss or death of a patient with a functioning graft. Graft loss was defined as the initiation of dialysis or the need for re-transplant. When a patient died with a functioning graft, this was considered as graft survival as the death was not due to renal problems. Transplantations in recipients under 19 years of age performed between 2005 and 2021 were included, using a complete case analysis. Patients with missing data were therefore excluded from the multivariate analysis. Other exclusion criteria comprised multiple organ transplantations, and transplantations performed outside Europe.

**Figure 1:**
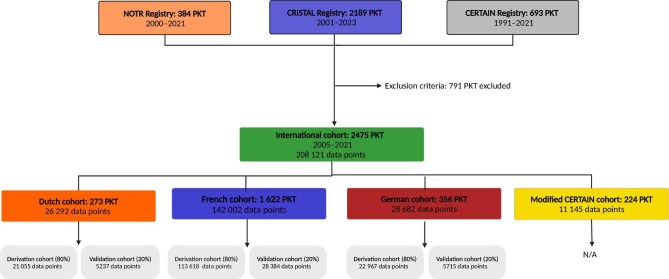
Flowchart of study data. Creation of cohorts used for development and analysis of predictive models. The modified CERTAIN cohort consisted of CERTAIN data excluding German, Dutch, and French data and was not used to develop a prediction model. CERTAIN: Cooperative European Pediatric Renal Transplant, NOTR: Nederlandse Orgaan Transplantatie Registratie.

Five cohorts were developed based on the available data (Fig. 1), including three national cohorts: Dutch, French, and German cohorts. A modified CERTAIN cohort was formed, incorporating the data from countries beyond the aforementioned ones (excluding non-European countries and transplantations before 2011). Furthermore, an international cohort combining all these datasets was used to develop an international prediction model.

The current external validation study used data from 2005 to 2021 onwards to ensure an adequately powered model that takes into account the substantial changes in health care over time. This decision was motivated by the scarcity of data in the external validation cohort. Therefore, an adapted ‘derivation cohort’ was developed based on 80% of the Dutch data on transplantations performed between 2005 and 2021.

### Statistical analysis

To address the primary objective, an external validation of the adapted Dutch model (2005–2021) was performed in the German and French cohorts. For the second objective, the international cohorts were used to re-estimate the regression coefficients of this Dutch model. As stated by Moons *et al*., it is recommended to adjust or update existing prediction models using the available external validation dataset by re-estimating the regression coefficients [16, 22].

Therefore, an adjusted prediction model was developed for the international cohort, as well as country-specific models for Germany and France, using the same methodology and identical predictors as the original model [14].

#### Model development

In summary, a person-period file was generated, treating each month of follow-up as a single observation point to account for the time-varying patterns of the determinants [23]. Each cohort was randomly divided into a derivation cohort (80%) and a validation cohort (20%).

The multivariable logistic regression model was fitted using the derivation cohorts and included the parameters mentioned before, with the time elapsed after transplantation modeled using restricted cubic splines [24, 25].

Natural splines with four nodes positioned at quantiles 0.05, 0.35, 0.65, and 0.95 were utilized, resulting in five time-related variables. The β-coefficients of this model were employed to construct a prediction score, predicting the probability of graft survival for each month after transplantation (Appendix 2, Tables 9–12).

To account for the different contribution rates of the individual countries within the international model, countries with fewer transplantations were weighted more heavily to equalize their influence. In addition, a dummy variable was created for each cohort.

#### Model performances

The quality of these models was evaluated by comparing the predicted survival probability of each model with the actual recorded outcome. Discriminative performance was assessed using the area under the curve (AUC) of the time-dependent receiver operating characteristics (ROC) curve for each year after transplantation in the validation cohorts [26]. Calibration in the validation cohorts was assessed using calibration plots and the Hosmer–Lemeshow test [22].

The prediction model was reported following the guidelines of the Transparent Reporting of a Multivariable Prediction Model for Individual Prognosis or Diagnosis (TRIPOD) statement (Appendix 3) [27].

Continuous baseline characteristics were summarized as means and standard deviations, or medians and interquartile ranges if the data deviated from normal distribution. Graft survival was evaluated using Kaplan–Meier curves and log-rank tests. Group comparisons were performed using chi-squared tests and one way ANOVA-tests, while the Mann–Whitney *U*-test was used for non-normally distributed data. Data analysis was performed using IBM SPSS Statistics v.29.0 and R v.4.3.1 [28, 29]. Statistical significance was set at *P *< .05. Graphics were created using BioRender.

## RESULTS

In total, the data from 3266 transplantations performed between 1991 and 2023 were collected from the different registries: two population-based national registries and one hospital-based European registry (Fig. 1). Among these, 2475 transplantations were included in the analysis, forming the international cohort. This international cohort was divided by country for the country-specific analysis, while the modified CERTAIN cohort exclusively comprised transplantations performed in other countries than Netherlands, France, or Germany.

**Figure 2:**
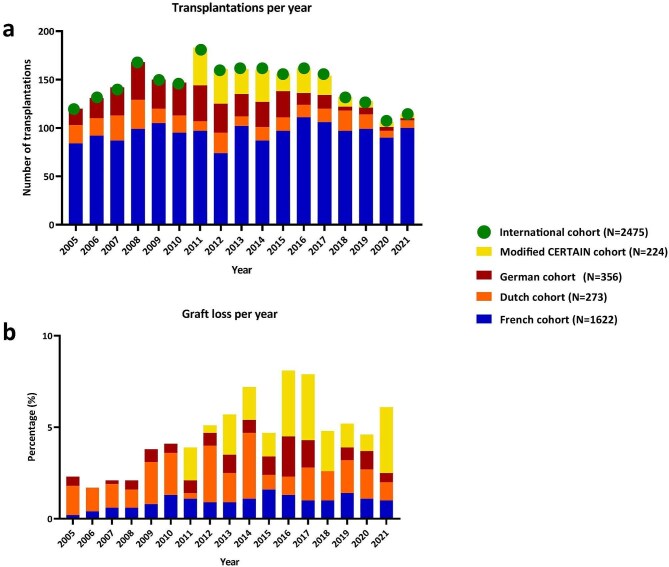
Annual transplantation rates. (**a**) Annual number of pediatric kidney transplantations per cohort. (**b**) The annual percentage of graft loss per cohort. The modified CERTAIN cohort consisted of CERTAIN data excluding German, Dutch, and French data.

**Figure 3:**
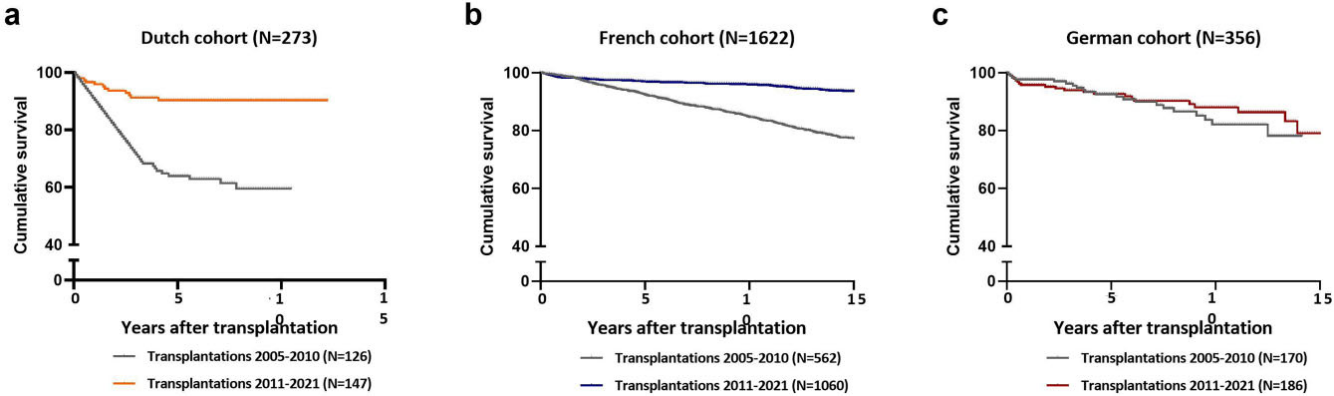
Graft survival across cohorts. Kaplan–Meier curves illustrating graft survival within each national cohort by era of transplantations. Detailed statistical comparisons between cohorts are provided in Appendix 1, Table 1. (**a**) Graft survival in the Dutch cohort per period of transplantation: *P* = .02. (**b**) Graft survival in the French cohort per period of transplantation: *P* = .02. (**c**) Graft survival in the German cohort per period of transplantation: *P* = .09.

The annual number of transplantations in the international cohort during the period 2005–2021 ranged from 106 to 183, most of which were performed in France (Fig. 2a). In addition, the percentage of annual graft loss varied between time periods and cohorts (Fig. 2b). These differences were likewise reflected in the survival curves of each cohort, with significant differences observed in transplantations performed in the first and second eras (Fig. 3). While graft survival significantly improved over time in the Dutch and French cohorts, this was not significant in the German cohort (Appendix 1, Table 1).

### Cohorts for prediction model development and validation

By excluding transplantations performed before 2005 and after 2021, a total of 2475 transplantations from four cohorts were eligible for analysis (Fig. 1, Table 1). There were significant differences in characteristics and outcome between the cohorts (Table 2). The Dutch cohort had a significantly higher number of living donations than both the French and German cohorts, and the donors in the Dutch cohort were significantly older. Detailed comparisons between cohorts can be found in Appendix 1, Table 2.

**Table 1:** Composition of the cohorts.

	Dutch cohort (*n* = 273)	French cohort (*n* = 1622)	German cohort (*n* = 356)	Modified CERTAIN cohort (*n* = 224)
Registry	NOTR		CERTAIN	CERTAIN
Transplantations per country % (*n*)				
Germany				
Greece				2 (5)
France				
Hungary				3 (7)
Ireland				18 (41)
Italy				24 (54)
The Netherlands		100 (1622)	100 (356)	
Poland				15 (34)
Russia				2 (4)
Spain	100 (273)			3 (7)
Switzerland				5 (12)
Turkey				10 (23)
United Kingdom				17 (37)

Data sources that were used to develop the different cohorts. The modified CERTAIN cohort consisted of CERTAIN data excluding the German, Dutch, and French transplantations.

**Table 2:** Cohort characteristics.

	International cohort (*n* = 2475)	Dutch cohort (*n* = 273)	French cohort (*n* = 1622)	German cohort (*n* = 356)	Modified CERTAIN cohort (*n* = 224)	*P* value	Test performed
Period PKT	2005–2021	2005–2021	2005–2021	2005–2021	2011–2021		
Follow-up (m) median [IQR]	76 [37–133]	94 [45–144]	85 [42–136]	68 [32–123]	39 [15–69]	**<** **.01**	Kruskall–Wallis
Graft loss % (*n*)	25 (418)	22 (61)	16 (262)	12 (42)	24 (53)	**<** **.01**	Chi^2^ test
Recipient age median [IQR]	13 [7–16]	11 [6–15]	13 [8–16]	11 [5–14]	13 [9–15]	**<** **.01**	Kruskall–Wallis
Donor age median [IQR]	19 [13–41]	43 [33–50]	16 [13–27]	35 [15–44]	34 [14–44]	**<** **.01**	Kruskall–Wallis
HLA MM median [IQR]	3 [2–4]	3 [2–3]	3 [3–4]	3 [2–3]	3 [2–4]	**<** **.01**	Kruskall–Wallis
LD % (*n*)	25 (619)	43 (116)	19 (308)	34 (122)	33 (73)	**<** **.01**	Chi^2^ test
Pre-emptive PKT % (*n*)	25 (608)	26 (71)	25 (401)	24 (84)	23 (52)	.87	Chi^2^ test
Re-transplant % (*n*)	8 (203)	13 (34)	7 (119)	10 (34)	7 (16)	**.03**	Chi^2^ test
Underlying disease % (*n*)							Chi2 test
CAKUT	36 (900)	29 (79)	37 (594)	41 (146)	36 (81)	**.02**	
Ciliopathy	9 (224)	8 (23)	8 (122)	14 (49)	13 (30)	**<** **.01**	
Glomerulopathy	16 (404)	11 (30)	16 (265)	19 (69)	18 (40)	**.04**	
Tubulopathy	4 (86)	1 (2)	4 (72)	2 (8)	2 (4)	**<** **.01**	
Microvascular thrombopathy	4 (110)	3 (9)	4 (70)	6 (22)	4 (9)	.32	
Hereditary nephropathy	7 (181)	9 (25)	9 (150)	1 (2)	2 (4)	**<** **.01**	
Metabolic nephropathy	2 (54)	2 (6)	2 (30)	3 (9)	4 (9)	.21	
Other underlying disease cause	21 (516)	36 (99)	20 (319)	14 (51)	21 (47)	**<** **.01**	

IQR, interquartile range.

Overview of the cohort characteristics with overall statistics, significance is highlighted in bold. Detailed statistical comparisons between cohorts are presented in the Appendix Table 4. The modified CERTAIN cohort consisted of CERTAIN data without the German, Dutch, and French data.

#### External validation of the Dutch prediction model

**Figure 4:**
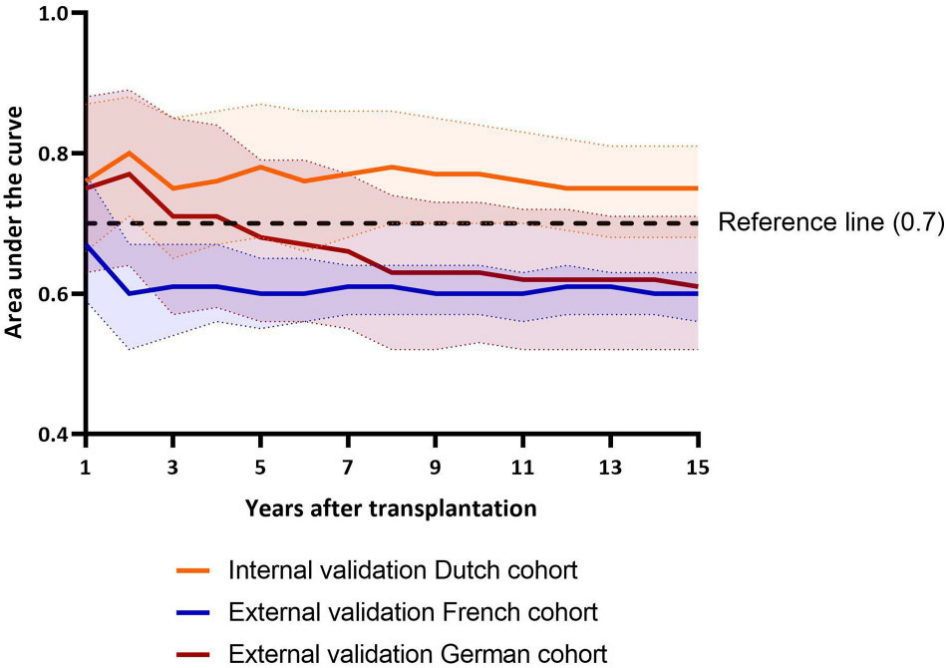
External validation of the Dutch prediction model. Predictive performance of a model based on the Dutch cohort. The model was externally validated in the French (*n* = 1622) and German (*n* = 356) cohorts and internally validated in the Dutch validation cohort. The predictive capacity was assessed by calculating the AUC for each year post-transplant, accompanied by a 95% confidence interval. A predictive capacity >0.7 was considered to be good.

The Dutch prediction model developed using the 80% derivation cohort (Appendix 1, Tables 4, and 9) showed good performance within the Dutch validation cohort (Fig. 4, orange line, Appendix Fig. 1) achieving an AUC of 0.76, 0.77, and 0.75 after 1, 10, and 15 years, respectively. However, external validation in the German and French cohorts resulted in a 10-year AUC of 0.63 and 0.60 (Fig. 4). In summary, the Dutch model showed good performance in the Dutch population, but exhibited limited predictive accuracy in other countries.

#### International model

**Figure 5:**
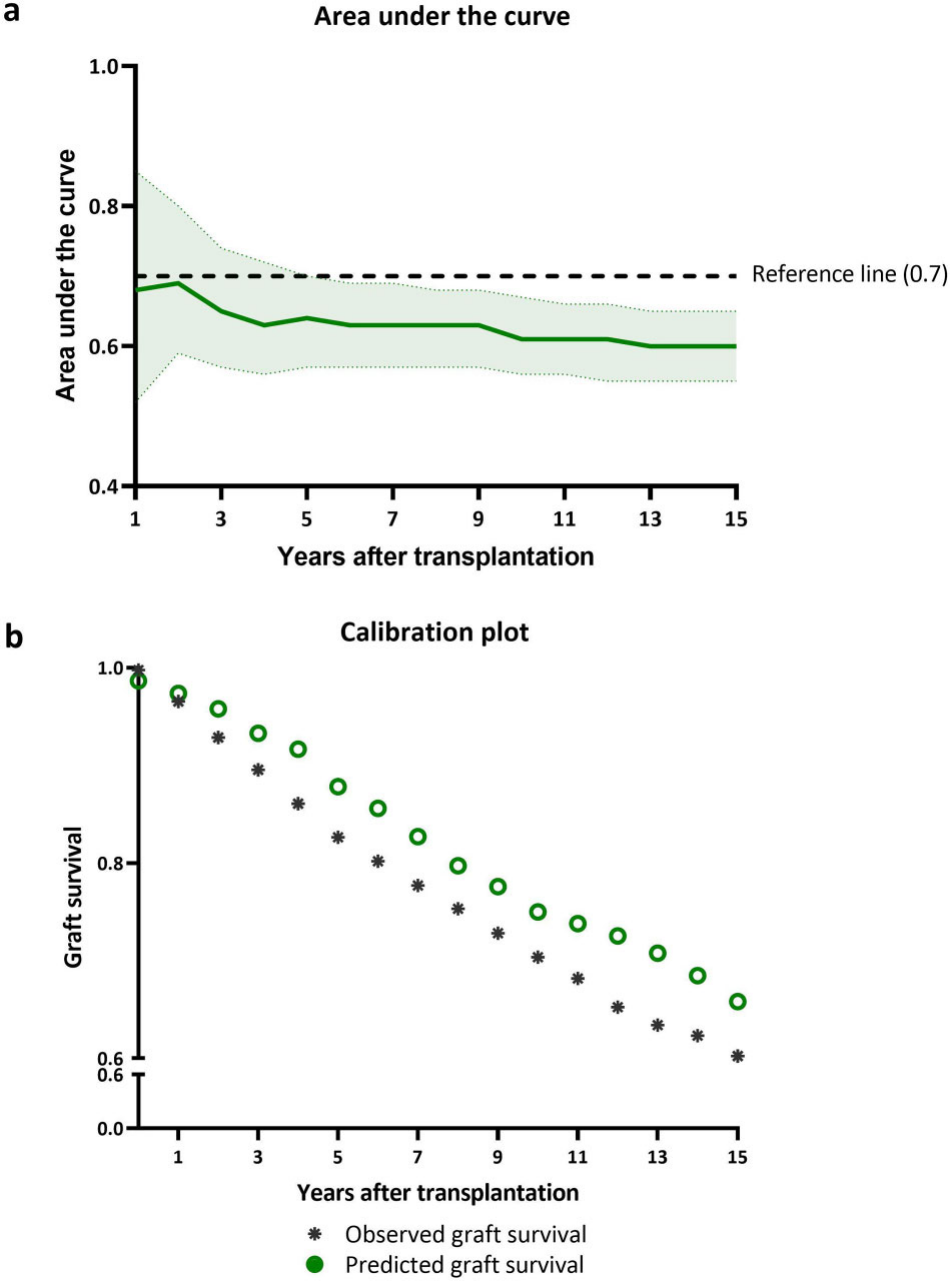
Predictive capacity international model. Predictive performance of a model derived from an international dataset of 2475 transplantations, where 80% of the data served as the derivation cohort. Characteristics of the derivation and validation cohorts are shown in the Appendix 1, Table 5. (**a**) AUC for each year after transplantation within the validation cohort with 95% confidence interval. (**b**) Calibration plot. Observed graft survival in the validation cohort and the graft survival predicted by the international prediction model. Hosmer–Lemeshow test: 12 119, (*P* = .15).

An international model was developed based on the 80% derivation cohort of all transplantations (Appendix Tables 5 and 12). Internal validation showed an AUC of 0.68, 0.61, and 0.60 at 1, 10, and 15 years (Fig. 5a) and poor calibration beyond 1-year post-transplantation (Fig. 5b) with overestimation of the true graft survival.

#### Predictive capacity national models

**Figure 6:**
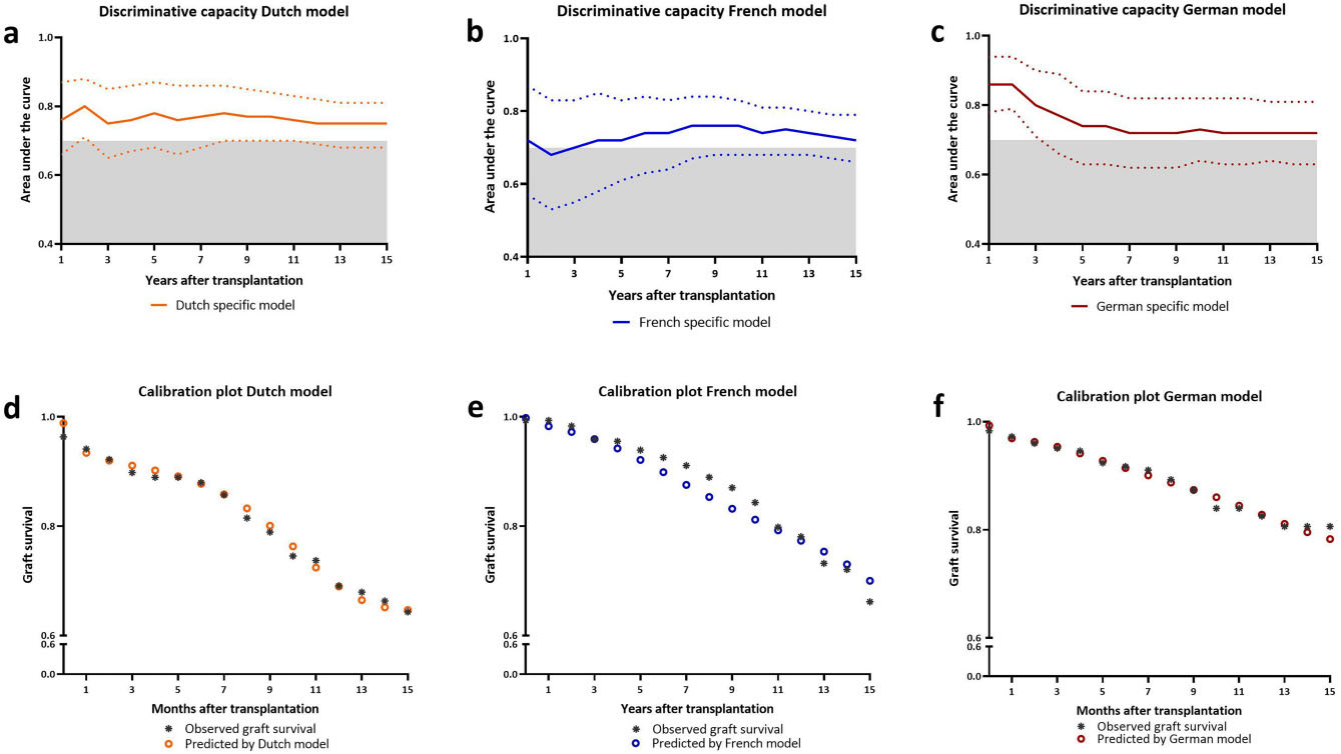
Predictive capacity national models. Internal validation of country-specific models assessing discrimination and calibration in the respective validation cohorts. (**a**) AUC for each year after transplantation for the Dutch model within the Dutch validation cohort with 95% CI. (**b**) AUC for each year after transplantation for the French model within the French validation cohort with 95% CI. (**c**) AUC for each year after transplantation for the German model within the German validation cohort with 95% CI. (**d**) Observed graft survival in the Dutch validation cohort compared to the graft survival predicted by the Dutch prediction model. Hosmer–Lemeshow test: 4668 (*P* = .79). (**e**) Observed graft survival in the French validation cohort versus graft survival predicted by the French prediction model. Hosmer–Lemeshow test: 9764 (*P* = .28). (**f**) Observed graft survival in the German validation cohort compared to graft survival predicted by the German prediction model Hosmer–Lemeshow test: 11 732 (*P* = .16).

To assess whether nationally derived models (Appendix Tables 6, 7, 10 and 11) performed better than an international model, internal validation was performed on the three different country-specific models (Fig. 6). The 10-year AUC was found to be 0.77, 0.76, and 0.73 in the Dutch, French, and German cohorts, respectively. Calibration plots showed a good fit within the first years after transplantation, although predictions became less accurate on the longer term. In summary, the national prediction models demonstrated strong predictive accuracy within their respective countries.

## DISCUSSION

This study reveals that, although the Dutch prediction model performs well within the Dutch population, its predictive accuracy diminishes significantly when applied to German or French cohorts. In addition, the model based on combined international data showed inferior predictive ability, despite being based on a larger database. This might be (partially) attributed to the heterogeneity of cohorts and predictor effects, both of which are influenced by differences in clinical care and protocols across countries.

### Cohort heterogeneity

The ‘modified CERTAIN cohort’ illustrates the heterogeneity of the European landscape. Preferably, an international model is based on the data of all those countries, however, the population and clinical practices seem to differ largely [31]. When the international model demonstrated limited predictive value, a country-specific approach was adopted. Country-specific tailored models had a good predictive ability in their own country. This discrepancy may be partly explained by differences in clinical practice between countries, as shown by differences in cohort characteristics.

Notable differences were observed in factors such as the use of living donors, the age of recipients and donors, and the prevalence of retransplants. Previous studies have also shown large differences in kidney allocation policies and transplantation practices among European countries [30, 31].

Moreover, there were notable disparities in the underlying diseases leading to kidney failure, such as the prevalence of patients with CAKUT. Similar variations in the reported number of patients with CAKUT have also been observed in other studies [32, 33]. These differences may be due to different classification systems and definitions. In addition, the utilization of prenatal screening could potentially contribute to the variations observed among countries [34, 35].

### Heterogeneity of predictor effects

External validation is an essential step in the development of prediction models, ensuring the reliability and applicability of a developed model beyond its initial dataset [16]. In general, the performance of prediction models is lower in an external cohort than in the derivation cohort. A well-established method for enhancing these models is to refine existing prediction models by incorporating information from the external validation datasets [16]. To improve both calibration and discriminative performance in the new cohort, it is recommended that all regression coefficients be re-estimated using data from the validation sample only [16, 36].

As expected, this geographical validation study revealed a decline in the predictive performance of the model when applied to external cohorts. Consequently, we adapted the model to these new datasets by developing country-specific models for each cohort, while also creating a generalized international model by re-estimating the regression coefficients. These updated coefficients differed significantly from the original model, highlighting the need for adaptation to ensure accurate predictions.

Heterogeneity in predictor effects is a well-known phenomenon and could be explained by several factors. Some of these discrepancies may be attributed to variations in cohort characteristics, as discussed previously [15]. For example, in the French cohort, more young deceased donors were used (*n* = 1064, 66%), particularly in contrast to the Dutch cohort (*n* = 33, 12%). This discrepancy may have influenced the divergent coefficients observed (negative in the Dutch model, positive in the French model) associated with a higher donor age. Besides, the higher rate of graft loss in the Dutch cohort can partly be attributed to the increased frequency of retransplants and a longer follow-up. However, other discrepancies, such as different estimates for time-related variables and intercepts, cannot be attributed solely to known cohort differences. This suggests that other differences in patient selection, protocols, or clinical practice may contribute to these differences.

Furthermore, several previously published prediction models, such as the iBOX, have also shown that various post-transplantation parameters significantly influence graft outcome [13, 37]. These factors are not included in the current model, and the diversity in post-transplant parameters may contribute to the heterogeneity of the regression coefficients. To enhance clinical applicability, this prediction model was intentionally designed to include only pre-transplantation variables. Including post-transplantation parameters might improve the international performance of the model. Furthermore, differences in transplantation outcome may also affect model performance, as our study showed a significant difference in graft survival between cohorts.

### Clinical applicability

As outlined in the original paper, this prediction model has the potential to support clinical decision-making regarding donor selection and transplantation strategies. We developed a risk calculation tool based on the original model that could be used in daily practice.

The tool estimates monthly graft survival based on the characteristics of both the patient and the potential donor, allowing for the comparison of multiple scenarios tailored to individual patients. For example, it can assist in evaluating the trade-off between waiting for a well-matched deceased donor while remaining on dialysis or proceeding with a pre-emptive transplant from a poorly matched LD [14].

While this tool is not intended to be the sole factor in decision-making and may be less impactful for patients with limited options, it contributes to personalized medicine by supporting nuanced clinical decisions. Additionally, it enhances communication with patients and their families, fostering shared decision-making [14].

To date, the Dutch model has primarily been implemented in Dutch clinics. To evaluate its influence on clinical decision-making, conducting a model impact study would be a valuable next step.

The utility of prediction models depends on their successful implementation and ongoing maintenance. The preference for country-specific models over a more generalized approach is a key finding of this study. It suggests that the significant diversity in clinical practice across Europe makes the uniform application of international prediction models a potential barrier.

Clinical decisions based on inaccurate prediction models could have major consequences for a wide range of patient outcomes, and the differences observed in the adapted models highlight the critical role of external validation.

Moreover, it is acknowledged that the performance of prediction models can deteriorate over time, a phenomenon known as “calibration drift” [38]. Given the continuous evolution of clinical practice and graft survival, it is essential to validate these models every few years to ensure their continued validity. However, the frequency of model updates may change over time.

In this study, graft survival showed significant changes over a 10-year period (Fig. 3), although we may expect less change in both clinical care and graft survival in the future. Innovative approaches, such as data-driven drift detection systems, may help to warn of deteriorating model performance and the need for updates [38, 39].

### Strengths and limitations

One of the strengths of this unique study in the field of PKT is its international collaboration and geographical diversity. The inclusion of data from several European centers offers a comprehensive perspective on clinical practices. In addition, the mandatory nature of the French and Dutch registries ensures the representativeness of these cohorts. Another strength is the comparison of country-specific models with an international model and the use of both internal and external validation.

Furthermore, the recent study by Coens *et al.* [10] underscores the significance of time-varying parameters. This study uniquely incorporates the influence of time on the relative hazards of these parameters by modeling time and integrating time-dependent variables.

Additionally, this study has direct clinical relevance, as these prediction models can enhance communication with patients and their families, as well as improve counseling on prognosis. It also provides the basis for further research to standardize and optimize clinical practice, with the possibility of developing a more accurate generalized prediction model.

#### Study limitations

An important limitation of the study is the selection of European centers and the inclusion of patients with complete data only, as these may not be fully representative of the entire European population. Moreover, a potential selection bias could exist within the German cohort due to the partially retrospective nature of the CERTAIN registry. While the Dutch and French datasets were considered representative of their countries, the German data were sourced from the CERTAIN registry, where data collection was not compulsory. Consequently, data from German centers not participating in CERTAIN might be lacking. However, the primary aim of this study was not to provide a comprehensive validation across all of Europe, but rather to explore the potential applicability of the model in different countries.

Second, we categorized the data by country, assuming uniform implementation of protocols throughout the country. However, clinical care can vary considerably within a single country due to regional differences and local practice. Unfortunately, in-depth analysis of centers and other countries was not possible due to limited sample size.

Third, the model could achieve better predictive performance by including a larger number of parameters, such as more detailed donor information or immunosuppressive regimen. However, the goal is to strike a balance between goodness of fit and parsimony. Using a reduced number of variables improves both the accuracy and the practicality of the model. The primary objective was to develop an easy-to-use pre-transplant prediction tool, rather than a prediction model that integrates post-transplant parameters.

Fourth, the quality of a registry depends on data accuracy and completeness. A significant number of patients were excluded due to missing data, and the transition to adulthood might have resulted in loss to follow-up in the voluntary registry, potentially introducing bias.

Finally, as mentioned before, clinical practice and patient populations may change over time, requiring regular updating of the current model. Including the factor ‘era of transplantation’ could enhance the model's accuracy, although it would add to the number of parameters.

### Future perspectives

The use of prediction models is increasing, driven by their potential to advance personalized medicine, individualized decision-making, and risk stratification. The field of PKT has lacked a prediction tool based solely on pre-transplant parameters. This model could serve as a basic template for other countries to develop their own nationally tailored models.

The results of this study show that there is considerable variation in clinical practice between European centers with regard to donor-recipient selection and transplant outcomes. At present, the impact of this diversity on transplant outcomes remains uncertain, and future research could assess whether current care would benefit from a more standardized approach. Should clinical practices in Europe become more standardized and comparable in the future, there may be opportunities to develop an international prediction model to benefit a wider range of patients.

However, large data sets are needed to develop adequately powered evidence-based guidelines. The establishment of international registries such as CERTAIN, the European Society for Pediatric Nephrology (ESPN)/ERA registry, and ERN-specific registries are valuable steps in this direction [17, 19, 40, 41]. However, this study also highlights the importance of comprehensive and accurate data collection from a representative cohort, rather than fragmented data collection. Therefore, the impact of these registries could be further enhanced through increased harmonization and collaboration.

## CONCLUSION

This external validation study showed that the Dutch pre-transplant risk assessment tool for PKT cannot be readily applied in other countries without country-specific adaptations. Due to the current diversity of clinical practice in Europe, a country-specific model is preferable to a generalized international model.

## Supplementary Material

sfaf031_Supplemental_File

## Data Availability

The raw data supporting the findings of this study are not owned by the authors but are held by the respective providing registries. Interested parties may request access to the raw data directly from the relevant registries. Please contact the corresponding author for assistance in facilitating such requests. Additionally, all relevant statistics can be found in the supplementary files.
