# Supplementary material for: International validation of a pre-transplant risk assessment tool for graft survival in pediatric kidney transplant recipients
Source: Clin Kidney J. 2025 Jan 28;18(3):sfaf031. doi: 10.1093/ckj/sfaf031 (PMC11883223; doi:10.1093/ckj/sfaf031)
Supplement: sfaf031_Supplemental_File [file sfaf031_supplemental_file.docx]

**Appendix 1- Supplementary Tables and Figures**

|  | **Dutch cohort 2005-2010 (N=126)** | **Dutch cohort 2011-2021 (N=147)** | **French cohort 2005-2010 (N=562)** | **French cohort 2011-2021 (N=1060)** | **German cohort**  **2005-2010 (N=170)** | **German cohort**  **2011-2021 (N=186)** |
| --- | --- | --- | --- | --- | --- | --- |
| **Dutch cohort**  **2005-2010 (N=126)** | **-** | **0.02** | 0.07 | **<0.01** | **<0.01** | 0.19 |
| **Dutch cohort**  **2011-2021 (N=147)** | **0.02** | **-** | 0.29 | 0.71 | 0.70 | 0.34 |
| **French cohort**  **2005-2010 (N=562)** | 0.07 | 0.29 | **-** | **0.02** | **0.01** | 0.63 |
| **French cohort**  **2011-2021 (N=1060)** | **<0.01** | 0.71 | **0.02** | - | 0.27 | **0.05** |
| **German cohort**  **2005-2010 (N=170)** | **<0.01** | 0.70 | **0.01** | 0.27 | - | 0.09 |
| **German cohort**  **2011-2021 (N=186)** | 0.19 | 0.34 | 0.63 | **0.05** | 0.09 | **-** |

**Supplementary table 1.** Statistical comparison graft survival across national cohorts. P-value of log-rank test between specific cohorts, significance highlighted in bold.

**Supplementary table 2. Statistical comparison characteristics across cohorts**. P-value of statistical test between specific cohorts. The modified CERTAIN cohort consisted of CERTAIN data without the German, Dutch and French transplantations. CAKUT: congenital anomalies of the kidney and urinary tract, IQR: interquartile range, HLA : human leucocyte antigen, KTx: kidney transplantation

|  | **Dutch vs French** | **Dutch vs**  **German** | **French vs German** | **Dutch vs mCERTAIN** | **German vs mCERTAIN** | **French vs mCERTAIN** | **Test performed** |
| --- | --- | --- | --- | --- | --- | --- | --- |
| **Graft survival by Kaplan Maier** | **0.04** | **0.02** | 0.30 | **<0.01** | **<0.01** | **<0.01** | **Log-rank** |
| **Months of follow-up** | 0.23 | **<0.01** | **<0.01** | **<0.01** | **<0.01** | **<0.01** | **KW** |
| **Graft loss** | **<0.20** | **<0.01** | **0.04** | 0.75 | **<0.01** | **0.01** | **Chi^2^ test** |
| **Recipient age** | **<0.01** | 0.21 | **<0.01** | **0.03** | **<0.01** | 0.21 | **KW** |
| **Donor age** | **<0.01** | **<0.01** | **<0.01** | **<0.01** | 0.63 | **<0.01** | **KW** |
| **HLA MM** | **<0.01** | 0.35 | **<0.01** | **0.04** | 0.21 | **<0.01** | **KW** |
| **Living donor** | **<0.01** | **0.04** | **<0.01** | **0.03** | 0.72 | **<0.01** | **Chi^2^ test** |
| **Pre-emptive KTx** | 0.65 | 0.51 | 0.68 | 0.53 | 1.00 | 0.68 | **Chi^2^ test** |
| **Retransplantation** | **0.01** | 0.25 | 0.16 | 0.05 | 0.36 | 1.00 | **Chi^2^ test** |
| **Underlying disease**  CAKUT  Ciliopathy  Glomerulopathy  Tubulopathy  Microvascular thrombopathy  Hereditary nephropathy  Metabolic nephropathy  Other underlying disease cause | **0.01**  0.62  **0.02**  **<0.01**  0.52  1.00  0.63  **<0.01** | **<0.01**  **0.04**  **<0.01**  0.20  0.14  **0.01**  1.00  **<0.01** | 0.13  **<0.01**  0.18  0.07  0.13  **<0.01**  0.40  **0.02** | 0.10  0.08  **0.04**  0.42  0.81  **<0.01**  0.30  **<0.01** | 0.26  1.00  0.66  0.77  0.34  0.21  0.33  **0.04** | 0.94  **0.01**  0.57  0.07  1.00  **<0.01**  **0.05**  0.66 | **Chi^2^ test** |

**Supplementary table 3. Characteristics per cohort per era.** Comparison of cohort characteristics of the Dutch, French and German cohorts before and after 2010. Significant differences between era’s within the respective cohorts are bold. CAKUT: congenital anomalies of the kidney and urinary tract, IQR: interquartile range, HLA : human leucocyte antigen, PKT: pediatric kidney transplantation

|  | **Dutch**  **2005-2010**  **(N=126)** | **Dutch**  **2011-2021 (N=147)** | **French**  **2005-2010 (N=562)** | **French**  **2011-2021 (N=1060)** | **German**  **2005-2010 (N=170)** | **German**  **2011-2021 (N=186)** |
| --- | --- | --- | --- | --- | --- | --- |
| **Months of follow-up, median [IQR]** | **152 [107-170]** | **60 [32-93]** | **158 [99-182]** | **62 [34-97]** | **106 [51-165]** | **50 [21-75]** |
| **Graft loss % (N)** | **38% (48)** | **9% (13)** | **31% (172)** | **9% (90)** | 12% (21) | 11% (21) |
| **Recipient age median [IQR]** | 12 [7-15] | 11 [5-15] | 13 [9-16] | 13 [8-16] | **9 [3-13]** | **13 [8-15]** |
| **Donor age median [IQR]** | 44 [34-51] | 42 [33-49] | 16 [12-26] | 16 [13-29] | **37 [23-43]** | **33 [12-44]** |
| **HLA MM median [IQR]** | 3 [2-3] | 3 [2-3] | 3 [3-4] | 3 [3-4] | **2 [2-3]** | **3 [2-4]** |
| **Living donor % (N)** | **30% (38)** | **53% (78)** | **16% (91)** | **21% (217)** | 36% (61) | 33% (61) |
| **Pre-emptive PKT % (N)** | 21% (27) | 30% (44) | 23% (129) | 26% (272) | 25% (42) | 23% (42) |
| **Retransplantation % (N)** | 13% (16) | 12% (18) | 7% (40) | 8% (79) | 10% (17) | 9% (17) |
| **Underlying disease % (N)**  CAKUT  Ciliopathy  Glomerulopathy  Tubulopathy  Microvascular thrombopathy  Hereditary nephropathy  Metabolic nephropathy  Other underlying disease cause | 34% (43)  6% (7)  10% (13)  1% (1)  6% (7)  7% (9)  2% (2)  35% (44) | 25% (36)  11% (16)  12% (17)  1% (1)  1% (2)  11% (16)  3% (4)  37% (55) | 34% (191)  8% (45)  **20% (113)**  4% (21)  4% (25)  **5% (30)**  2% (11)  **22% (126)** | 38% (403)  7% (77)  **14% (152)**  5% (51)  4% (45)  **11% (120)**  2% (19)  **18% (193)** | 41% (70)  13% (22)  19% (32)  2% (4)  7% (11)  1% (1)  3% (5)  15% (25) | 41% (76)  15% (27)  20% (37)  2% (4)  6% (11)  1% (1)  2% (4)  14% (26) |

**Supplementary table 4.** **Characteristics Dutch derivation (80%) and validation (20%) cohort**. CAKUT: congenital anomalies of the kidney and urinary tract, IQR: interquartile range, HLA : human leucocyte antigen

|  | **Derivation cohort**  **(N=21055 data points )** | | **Validation cohort**  **(N=5237 datapoints)** | ***P*-value** |
| --- | --- | --- | --- | --- |
| **Period transplantations** | | 2005-2021 | 2005-2021 |  |
| **Months of follow-up median [IQR]** | | 137 [96-168] | 137 [95-166] | 0.89 |
| **Recipient age median [IQR]** | | 11 [6-15] | 11 [6-15] | 0.17 |
| **Donor age median [IQR]** | | 43 [34-50] | 43 [34-50] | 0.46 |
| **HLA MM median [IQR]** | | 3 [2-3] | 3 [2-3] | 0.31 |
| **Living donor % (N)** | | 39 (8306) | 19 (2041) | 0.54 |
| **Pre-emptive transplantation % (N)** | | 26 (5473) | 26 (1359) | 0.96 |
| **Retransplantation % (N)** | | 13 (2635) | 13 (666) | 0.69 |
| **Underlying disease % (N)**  CAKUT  Ciliopathy  Glomerulopathy  Tubulopathy  Microvascular thrombopathy  Hereditary nephropathy  Metabolic nephropathy  Other underlying disease cause | | 33 (6863)  7 (1459)  8 (1714)  1(240)  4 (896)  9 (1918)  2 (939)  36 (7572) | 32 (1695)  7 (380)  8 (434)  1(53)  4 (209)  10 (527)  2 (89)  35 (1850) | 0.75  0.41  0.74  0.46  0.42  **0.04**  0.45  0.39 |

**Supplementary table 5. Characteristics international derivation and validation cohort**. CAKUT: congenital anomalies of the kidney and urinary tract, IQR: interquartile range, HLA : human leucocyte antigen.

|  | **Derivation cohort**  **(N=166254 data points )** | **Validation cohort**  **(N=41868 datapoints)** | **P-value** |
| --- | --- | --- | --- |
| **Period transplantations** | 2005-2021 | 2005-2021 |  |
| **Months of follow-up median [IQR]** | 130 [85-169] | 131 [85-169] | 0.13 |
| **Recipient age median [IQR]** | 12 [7-15] | 12 [7-15] | 0.67 |
| **Donor age median [IQR]** | 18 [13-39] | 18 [13-40] | 0.11 |
| **HLA MM median [IQR]** | 3 [2-4] | 3 [2-4] | 0.09 |
| **Living donor % (N)** | 23 (38465) | 23 (9764) | 0.43 |
| **Pre-emptive transplantation % (N)** | 27 (45274) | 27 (11388) | 0.90 |
| **Retransplantation % (N)** | 8 (12798) | 8 (3225) | 0.98 |
| **Underlying disease % (N)**  CAKUT  Ciliopathy  Glomerulopathy  Tubulopathy  Microvascular thrombopathy  Hereditary nephropathy  Metabolic nephropathy  Other underlying disease cause | 37 (62176)  9 (14977)  17 (28180)  4 (6127)  5 (8306)  3 (5339)  2 (3727)  23 (37421) | 37 (15513)  9 (3780)  17 (7157)  4 (1522)  5 (2083)  3 (1384)  2 (973)  23 (9456) | 0.19  0.90  0.49  0.63  0.87  0.33  0.31  0.73 |

**Supplementary table 6. Characteristics French derivation and validation cohort**. CAKUT: congenital anomalies of the kidney and urinary tract,IQR: interquartile range, HLA : human leucocyte antigen

|  | **Derivation cohort**  **(N=113618 datapoints )** | **Validation cohort**  **(N=28384 datapoints)** | **P-value** |
| --- | --- | --- | --- |
| **Period transplantations** | 2005-2021 | 2005-2021 |  |
| **Months of follow-up median [IQR]** | 133 [87-170] | 133 [86-170] | 0.41 |
| **Recipient age median [IQR]** | 13 [8-16] | 13 [8-16] | 0.27 |
| **Donor age median [IQR]** | 16 [12-24] | 16 [12-24] | 0.36 |
| **HLA MM median [IQR]** | 3 [3-4] | 3 [3-4] | 0.84 |
| **Living donor % (N)** | 17 (18746) | 17 (4721) | 0.59 |
| **Pre-emptive transplantation % (N)** | 28 (31656) | 28 (7814) | 0.27 |
| **Retransplantation % (N)** | 7 (7735) | 7 (1871) | 0.20 |
| **Underlying disease % (N)**  CAKUT  Ciliopathy  Glomerulopathy  Tubulopathy  Microvascular thrombopathy  Hereditary nephropathy  Metabolic nephropathy  Other underlying disease cause | 37 (42234)  8 (9263)  19 (21181)  5 (5439)  5 (5709)  3 (3214)  2 (2687)  21 (23891) | 37 (10449)  8 (2323)  19 (5413)  5 (1305)  5 (1406)  3 (742)  2 (650)  21 (6096) | 0.27  0.87  0.10  0.19  0.64  0.06  0.47  0.10 |

**Supplementary table 7. Characteristics German derivation and validation cohort**. CAKUT: congenital anomalies of the kidney and urinary tract, IQR: interquartile range, HLA : human leucocyte antigen

|  | **Derivation cohort**  **(N=22967 data points )** | **Validation cohort**  **(N=5715 datapoints)** | **P-value** |
| --- | --- | --- | --- |
| **Period transplantations** | 2005-2021 | 2005-2021 |  |
| **Months of follow-up median [IQR]** | 123 [80-170] | 123 [75-170] | 0.89 |
| **Recipient age median [IQR]** | 8 [3-12] | 8 [3-12] | 0.77 |
| **Donor age median [IQR]** | 34 [16-43] | 34 [16-43] | 0.96 |
| **HLA MM median [IQR]** | 3 [2-3] | 3 [2-3] | 0.46 |
| **Living donor % (N)** | 35 (7981) | 19 (1972) | 0.73 |
| **Pre-emptive transplantation % (N)** | 26 (5909) | 26 (1474) | 0.50 |
| **Retransplantation % (N)** | 8 (1843) | 8 (474) | 0.50 |
| **Underlying disease % (N)**  CAKUT  Ciliopathy  Glomerulopathy  Tubulopathy  Microvascular thrombopathy  Hereditary nephropathy  Metabolic nephropathy  Other underlying disease cause | 42 (9699)  14 (3202)  18 (4199)  2 (416)  6 (1386)  0 (99)  2 (481)  15 (3485) | 43 (2458)  13 (3202)  19 (1061)  2 (97)  6 (321)  0 (17)  2 (123)  16 (892) | 0.29  0.08  0.62  0.61  0.25  0.17  0.80  0.41 |

**Supplementary table 8. Classification of underlying diseases.** CAKUT: congenital anomalies of the kidney and urinary tract

| **Primary renal disease** | **Classification** |
| --- | --- |
| **Amyloidosis** | Glomerulopathy |
| **Chronic renal failure - etiology uncertain** | Other |
| **Congenital renal dysplasia with/without urinary tract malformation** | CAKUT |
| **Congenital renal hypoplasia – Type unspecified** | CAKUT |
| **Crescentic (extracapillary) glomerulonephritis (type I, II, III)** | Glomerulopathy |
| **Cystic kidney disease - Type unspecified** | CAKUT |
| **Cystinosis** | Metabolic Nephropathy |
| **Dense deposit disease MPGN - Type II** | Glomerulopathy |
| **Denys-Drash syndrome** | Glomerulopathy |
| **Diabetes glomerulosclerosis or diabetic nephropathy - Type I** | Other |
| **Focal segmental glomerulosclerosis with nephrotic syndrome in adults** | Glomerulopathy |
| **Focal segmental glomerulosclerosis with nephrotic syndrome in children** | Glomerulopathy |
| **Glomerulonephritis - histologically examined** | Glomerulopathy |
| **Glomerulonephritis - histologically not examined** | Glomerulopathy |
| **Goodpasture's syndrome** | Glomerulopathy |
| **Hemolytic Uremic Syndrome including Moschcowitz syndrome** | Microvascular thrombopathy |
| **Henoch-Schonlein Purpura** | Microvascular thrombopathy |
| **Hereditary nephritis with nerve deafness (Alport's syndrome)** | Glomerulopathy |
| **Hereditary/Familial nephropathy – Type unspecified** | Hereditary nephropathy |
| **IgA nephropathy (proven by immunofluorescence)** | Other |
| **Joubert syndrome** | Ciliopathy |
| **Interstitial nephritis (not pyelonephritis) due to other cause, or unspecified** | Tubulopathy |
| **Iscemic renal disease / cholesterol embolism** | Other |
| **Kidney tumor** | Glomerulopathy |
| **Lupus Erythematosus** | Ciliopathy |
| **Medullary Cystic Disease – including nephronophthisis** | Ciliopathy |
| **Membrano-proliferative glomerulonephritis -Type I** | Glomerulopathy |
| **Membranous nephropathy** | Glomerulopathy |
| **Nephrocalcinosis and hypercalcaemic nephropathy** | Metabolic nephropathy |
| **Nephropathy caused by other specific drug** | Other |
| **Nephropathy due to Cyclosporin A** | Tubulopathy |
| **Oligomeganephronic hypoplasia** | CAKUT |
| **Other identified renal disorders - Specify** | Other |
| **Polycystic Kidneys - Adult type (dominant)** | Ciliopathy |
| **Polycystic Kidneys - Infantile type (recessive)** | Ciliopathy |
| **Primary oxalosis** | Metabolic nephropathy |
| **Pyelonephritis associated with neurogenic bladder** | CAKUT |
| **Pyelonephritis due to vesico-ureteric reflux without obstruction** | CAKUT |
| **Pyelonephritis/Interstitial nephritis - Acquired obstructive uropathy** | CAKUT |
| **Renal hypoplasia (congenital)** | CAKUT |
| **Syndrome of agenesis of abdominal muscles (Prune Belly)'** | CAKUT |
| **Tubular necrosis (irreversible)** | Tubulopathy |

**Appendix 2 – prediction models**

**Calculation of the prediction score in the derivation cohort.**

The prediction score was based on the multivariable logistic regression analyses and can be calculated for every month after transplantation, as can the graft survival. The weighted coefficients for the variables associated with graft loss as well as the B-coefficients derived from the modelling of time (time variable 1-5). These time variables are specific for each month after transplantation

| **Cohort** | **Dutch cohort** |
| --- | --- |
| **Source** | NOTR, Dutch transplantations |
| **Derivation cohort** | 21055 datapoints |
| **Formula prediction score** | $Prediction score=$  $Recipient age*0.051258951840646+$  $Donor age*-0.00166990251067+$  $Total HLA mismatches*0.033338001281215+$  $HLA-DR mismatches*-0.191657203376586+$  $Living donor*-0.307888675358515+$  $Time variable 1*-2.913001869388022+$  $Time variable 2*-1.548777547620199+$  $Time variable 3*0.962219148375916+$  $Time variable 4*-5.507652079021607+$  $Time variable 5*-3.801854253856693+$  $CAKUT*1.31161660250551+$  $Ciliopathy*1.423148145216895+$  $Tubulopathy*-12.991299547635297+$  $Metabolic nephropathies*1.87237526190387+$  $Glomerulopathy*2.750343895335345+$  $Microvascular Thrombopathy*1.617017706633996+$  $Other underlying disease*1.423148145216895+$  $Pre-emptive transplantation*-0.366367462692929+$  $Retransplantation*0.455748949752823+$  $Microvascular Thrombopathy*0+$  $Transplantation in the Netherlands*1+$  $Transplantation in Germany*0+$  $Transplantation in France*0+$  *Transplantation in CERTAIN cohort*0+*  *-6.434348358851875.* |
| **Formula graft survival** | $\boldsymbol{Graft survival=1-}\frac{\boldsymbol{e}^{\boldsymbol{prediction score}}}{\boldsymbol{1+}\boldsymbol{e}^{\boldsymbol{prediction score}}}$ |

**Supplementary table 9. Model development Dutch prediction model.** Description of the formulas and regression coefficients used to develop the Dutch prediction model.

**Supplementary table 10. Model development French prediction model.** Description of the formulas and regression coefficients used to develop the French prediction model.

| **Cohort** | **French cohort** |
| --- | --- |
| **Source** | Cristal, French transplantations |
| **Derivation cohort** | 113618 datapoints |
| **Formula prediction score** | *Prediction score =*  *Recipient age*0.114992972177554+*  *Donor age*0.018189008855287+*  *Total HLA mismatches*-0.132639852065916+*  *HLA-DR mismatches*0.247448105528644+*  *Living donor*-0.4502736732625035+*  *Time variable 1*-0.136416497839555+*  *Time variable 2*0.415855022617495+*  *Time variable 3*-0.007881105586925+*  *Time variable 4*0.348569257005142+*  *Time variable 5*2.792334895633161+*  *CAKUT*0.160273437309807+*  *Ciliopathy*-0.059519004508939+*  *Tubulopathy*-0.333144575228877+*  *Metabolic nephropathies*-1.496786546770948+*  *Glomerulopathy*-0.190931264261209+*  *Microvascular Thrombopathy*-0.460359489375019+*  *Other underlying disease*0.020536156822197+*  *Pre-emptive transplantation*-0.62017358184524+*  *Retransplantation*0.218650718785208+*  *Transplantation in the Netherlands*0+*  *Transplantation in Germany*0+*  *Transplantation in France*1+*  *Transplantation in CERTAIN cohort*0+*  *-7.720434926157283.* |
| **Formula graft survival** | $\boldsymbol{Graft survival=1-}\frac{\boldsymbol{e}^{\boldsymbol{prediction score}}}{\boldsymbol{1+}\boldsymbol{e}^{\boldsymbol{prediction score}}}$ |

**Supplementary table 11. Model development German prediction model.** Description of the formulas and regression coefficients used to develop the German prediction model.

| **Cohort** | **German cohort** |
| --- | --- |
| **Source** | CERTAIN, German transplantations |
| **Derivation cohort** | 22967 datapoints |
| **Formula prediction score** | *Prediction score =*  *Recipient age*0.042493471601405+*  *Donor age*0.003186807101762+*  *Total HLA mismatches*0.017360865182964+*  *HLA-DR mismatches*0.158488956760932+*  *Living donor*-0.309704705658886+*  *Time variable 1*-1.444591720525368+*  *Time variable 2*-1.960773161685392+*  *Time variable 3*0.428739637346107+*  *Time variable 4*-4.300375913967946+*  *Time variable 5*-0.219595492969816+*  *CAKUT*14.891105996428978+*  *Ciliopathy*15.042232270513493+*  *Tubulopathy*15.969879658887544+*  *Metabolic nephropathies*0.19677726042263+*  *Glomerulopathy*15.0204693230055+*  *Microvascular Thrombopathy*14.159022945125834+*  *Other underlying disease*13.997883129844753+*  *Pre-emptive transplantation*-1.117473998661477+*  *Retransplantation*0.670772130078361+*  *Transplantation in the Netherlands*0+*  *Transplantation in Germany*1+*  *Transplantation in France*0+*  *Transplantation in CERTAIN cohort*0+*  *-20.360814443242102.* |
| **Formula graft survival** | $\boldsymbol{Graft survival=1-}\frac{\boldsymbol{e}^{\boldsymbol{prediction score}}}{\boldsymbol{1+}\boldsymbol{e}^{\boldsymbol{prediction score}}}$ |

**Supplementary table 12. Model development international prediction model.** Description of the formulas and regression coefficients used to develop the international prediction model.

| **Cohort** | **All data** |
| --- | --- |
| **Source** | CERTAIN, NOTR, CRISTAL |
| **Derivation cohort** | 166254 data points |
| **Formula prediction score** | *Prediction score =*  *Recipient age*0.07019566420873+*  *Donor age*2.0216E-7+*  *Total HLA mismatches*-0.040009610013697+*  *HLA-DR mismatches*0.23390788252478+*  *Living donor*-0.251230667961937+*  *Timevariable1*-1.392086803986499+*  *Timevariable2*-1.446042032400365+*  *Timevariable3*-0.130357648747275+*  *Timevariable4*-2.923902116646464+*  *Timevariable5*1.645476314936974+*  *CAKUT*0.445511753467505+*  *Ciliopathy*0.367707218593693+*  *Tubulopathy*-0.213363332197728+*  *Metabolic nephropathies*0.053377024989259+*  *Glomerulopathy*0.474052502991594+*  *Hereditary nephropathy*15.437599620642201+*  *Microvascular Thrombopathy*-0.008364474672198+*  *Other underlying disease*0.110165607414321+*  *Pre-emptive transplantation*-0.675895541547728+*  *Retransplantation*0.414137326231995+*  *Transplantation in the Netherlands*-0.830858417739408+*  *Transplantation in Germany*-1.097085396901002+*  *Transplantation in France*-1.174132528263654+*  *Transplantation in CERTAIN cohort*0+*  *-4.941340549801808.* |
| **Formula graft survival** | $\boldsymbol{Graft survival=1-}\frac{\boldsymbol{e}^{\boldsymbol{prediction score}}}{\boldsymbol{1+}\boldsymbol{e}^{\boldsymbol{prediction score}}}$ |

**Appendix 3 - TRIPOD statement**

| **Section/Topic** | **Item** | **Checklist Item** | **Page** |
| --- | --- | --- | --- |
| **Title and abstract** | | | |
| Title | 1 | Identify the study as developing and/or validating a multivariable prediction model, the target population, and the outcome to be predicted. | 1 |
| Abstract | 2 | Provide a summary of objectives, study design, setting, participants, sample size, predictors, outcome, statistical analysis, results, and conclusions. | 2 |
| **Introduction** | | | |
| Background and objectives | 3a | Explain the medical context (including whether diagnostic or prognostic) and rationale for developing or validating the multivariable prediction model, including references to existing models. | 3 |
|  | 3b | Specify the objectives, including whether the study describes the development or validation of the model or both. | 3 |
| **Methods** | | | |
| Source of data | 4a | Describe the study design or source of data (e.g., randomized trial, cohort, or registry data), separately for the development and validation data sets, if applicable. | 4 |
|  | 4b | Specify the key study dates, including start of accrual; end of accrual; and, if applicable, end of follow-up. | 5 |
| Participants | 5a | Specify key elements of the study setting (e.g., primary care, secondary care, general population) including number and location of centres. | 4-5 |
|  | 5b | Describe eligibility criteria for participants. | 5 |
|  | 5c | Give details of treatments received, if relevant. | N/A |
| Outcome | 6a | Clearly define the outcome that is predicted by the prediction model, including how and when assessed. | 4-5 |
|  | 6b | Report any actions to blind assessment of the outcome to be predicted. | N/A |
| Predictors | 7a | Clearly define all predictors used in developing or validating the multivariable prediction model, including how and when they were measured. | 4-5 |
|  | 7b | Report any actions to blind assessment of predictors for the outcome and other predictors. | N/A |
| Sample size | 8 | Explain how the study size was arrived at. | 4-5 |
| Missing data | 9 | Describe how missing data were handled (e.g., complete-case analysis, single imputation, multiple imputation) with details of any imputation method. | 5 |
| Statistical analysis methods | 10a | Describe how predictors were handled in the analyses. | 5 |
|  | 10b | Specify type of model, all model-building procedures (including any predictor selection), and method for internal validation. | 5-6 |
|  | 10d | Specify all measures used to assess model performance and, if relevant, to compare multiple models. | 6 |
| Risk groups | 11 | Provide details on how risk groups were created, if done. | N/A |
| **Results** | | | |
| Participants | 13a | Describe the flow of participants through the study, including the number of participants with and without the outcome and, if applicable, a summary of the follow-up time. A diagram may be helpful. | 7 |
|  | 13b | Describe the characteristics of the participants (basic demographics, clinical features, available predictors), including the number of participants with missing data for predictors and outcome. | 9-11 Appendix table 1-2 |
| Model development | 14a | Specify the number of participants and outcome events in each analysis. | Appendix table 3-6 |
|  | 14b | If done, report the unadjusted association between each candidate predictor and outcome. | N/A |
| Model specification | 15a | Present the full prediction model to allow predictions for individuals (i.e., all regression coefficients, and model intercept or baseline survival at a given time point). | Appendix table 8-11 |
|  | 15b | Explain how to the use the prediction model. | Appendix 10 |
| Model performance | 16 | Report performance measures (with CIs) for the prediction model. | 12-15 |
| **Discussion** | | | |
| Limitations | 18 | Discuss any limitations of the study (such as nonrepresentative sample, few events per predictor, missing data). | 18 |
| Interpretation | 19b | Give an overall interpretation of the results, considering objectives, limitations, and results from similar studies, and other relevant evidence. | 18-19 |
| Implications | 20 | Discuss the potential clinical use of the model and implications for future research. | 19 |
| **Other information** | | | |
| Supplementary information | 21 | Provide information about the availability of supplementary resources, such as study protocol, Web calculator, and data sets. | Appendix |
| Funding | 22 | Give the source of funding and the role of the funders for the present study. | N/A |
